# Impact of *Bifidobacterium longum* Subspecies *infantis* on Pediatric Gut Health and Nutrition: Current Evidence and Future Directions

**DOI:** 10.3390/nu16203510

**Published:** 2024-10-16

**Authors:** Vanessa Nadia Dargenio, Fernanda Cristofori, Viviana Fara Brindicci, Federico Schettini, Costantino Dargenio, Stefania Paola Castellaneta, Andrea Iannone, Ruggiero Francavilla

**Affiliations:** 1 Interdisciplinary Department of Medicine, Pediatric Section, Children’s Hospital ‘Giovanni XXIII’, University of Bari “Aldo Moro”, 70126 Bari, Italy; vanessa.dargenio@unifg.it (V.N.D.); fernandacristofori@gmail.com (F.C.); v.brindicci1@studenti.uniba.it (V.F.B.); costy.dargenio@gmail.com (C.D.); castellanetas@gmail.com (S.P.C.); 2 Neonatology and Neonatal Intensive Care, Santissima Annunziata Hospital, 74123 Taranto, Italy; federico.schettini@uniba.it; 3 Gastroenterology Unit, Department of Emergency and Organ Transplantation, University of Bari “Aldo Moro”, 70126 Bari, Italy; ianan@hotmail.it

**Keywords:** Bifidobacterium, *Bifidobacterium longum* subspecies *infantis*, probiotics, gut, microbiota, children, infants

## Abstract

**Background:** the intestinal microbiota, a complex community vital to human health, is shaped by microbial competition and host-driven selective pressures. Among these microbes, *Bifidobacterium* plays a crucial role in early gut colonization during neonatal stages, where *Bifidobacterium longum* subspecies *infantis* (*B. infantis*) predominates and is particularly prevalent in healthy breastfed infants. **Objectives:** as we embark on a new era in nutrition of the pediatric population, this study seeks to examine the existing understanding regarding *B. infantis*, encompassing both preclinical insights and clinical evidence. **Methods:** through a narrative disceptation of the current literature, we focus on its genetic capacity to break down various substances that support its survival and dominance in the intestine. **Results:** using “omics” technologies, researchers have identified beneficial mechanisms of *B. infantis*, including the production of short-chain fatty acids, serine protease inhibitors, and polysaccharides. While *B. infantis* declines with age and in various diseases, it remains a widely used probiotic with documented benefits for infant and child health in numerous studies. **Conclusions:** the current scientific evidence underscores the importance for ongoing research and clinical trials for a deeper understanding of *B. infantis*’s role in promoting long-term health.

## 1. Introduction

Lactobacilli and bifidobacteria are commensal bacteria residing in the gastrointestinal (GI) tracts of humans and animals. From these genera, various strains are extensively included in dietary supplements, fermented products, and used as probiotics. Despite making up only a minor fraction of the mature and complex microbiome in adults, the significance of lactobacilli is underscored by evidence linking their modulation to various diseases.

Bifidobacteria are recognized as among the earliest colonizers of the GI tract in healthy infants, predominating until the introduction of a solid food diet, at which point the diversity of microbiome expands towards the complex composition typically observed in adults.

Over the past few decades, probiotics have been scrutinized for their purported health benefits, particularly their ability to influence the gut microbiome. However, recent research has cast doubt on the efficacy of probiotics in adults, with studies suggesting only minimal and short-lived impacts on GI microbial composition and function. This skepticism has prompted widespread questioning of the therapeutic potential of probiotics across various health contexts.

As awareness of the microbiome’s role in health and diseases of the pediatric population grows, probiotics are increasingly regarded as a promising intervention to modulate the microbiome in children. In contrast to the uncertainties surrounding adult probiotic use, recent studies have underscored a promising role for specific strains of the *Bifidobacterium longum* subspecies *infantis* (*B. infantis*) in shaping the GI microbiome of infants. These findings challenge the prevailing doubts by demonstrating profound and lasting effects on the GI composition of microbes and host health in early stages of life.

The development of a balanced GI microbiota in early life profoundly influences long-term health outcomes. Among the microbial communities acquired during infancy, bifidobacteria are acknowledged as crucial residents of the human GI microbiota, offering vital roles in the health of the pediatric population [1]. They were first identified in the stool of breastfed (BF) infants in the late 19th century [2]; bifidobacteria are currently known to quickly colonize the infant gut shortly after birth, which can often make up as much as 80% of the entire GI microbiota [3,4].

Maternal vertical transmission, either through vaginal delivery, GI tract, or breast milk, is crucial in seeding the infant’s GI tract with bifidobacteria, highlighting the critical importance of early microbial exposure in shaping gut colonization dynamics [5]. The modality of delivery significantly influences the infant’s initial exposure to microbes, which in turn influences the colonization of their intestine. Vaginal birth generally introduces beneficial microbes from the mother, such as *Bifidobacterium* spp., to the neonate. Conversely, cesarean delivery may slow down the process of bacterial colonization and alter the infant’s GI microbiome composition [6]. Moreover, antibiotic use may interfere with the fragile balance of the still-developing GI microbiome, while infant formula typically lacks the specific oligosaccharides present in breast milk that support the proliferation of a good microbial population.

Antibiotics can disturb the fragile balance of the developing gut microbiome, and infant formula usually does not contain the unique oligosaccharides found in breast milk that encourage the growth of beneficial bacteria [7,8].

The prevalence of *Bifidobacterium species* (spp.), particularly *B. infantis*, in the infant GI tract has been linked to numerous health benefits. These include the acceleration of immune system maturation, modulation of immune responses to suppress inflammation, enhancement of the GI barrier function, and increased production of beneficial metabolites like acetate. Reduced levels of bifidobacteria in infants have been linked to chronic medical conditions such as asthma, obesity [9], as well as reduced vaccine efficacy [10] and autoimmune and allergic diseases more common in wealthier countries [11]. A decline in bifidobacteria, along with a rise in pro-inflammatory microorganisms during infancy, is thought to coincide with a key period in immune system development, potentially heightening the risk of immune-related conditions later in life. Current studies on *Bifidobacterium* spp. have revealed their complex roles in the GI ecosystem, where they engage in both microbial and host interactions [12,13]. This symbiotic relationship between *B. infantis* and the developing infant gut exemplifies a coevolutionary adaptation aimed at nurturing a healthy GI microbiome during the crucial preweaning period [14].

*B. infantis* is a key member of the human GI microbiota and is commonly employed in probiotic formulations. Recent investigations have shown that supplementation with specific strains of *B. infantis* can lead to significant increases in their abundance within the infant gut [15]. For instance, studies have reported a remarkable ten million-fold increase in fecal *B. infantis* levels following its administration, persisting for extended periods even after cessation of supplementation [16]. Moreover, infants receiving *B. infantis* supplementation exhibited reduced levels of potential gut pathogens, decreased GI inflammation, and fewer antibiotic resistance genes compared with their non-supplemented counterparts [15]. These findings suggest a potential therapeutic avenue for enhancing infant GI health and mitigating disease risk early in life. In light of these compelling findings, there is growing interest in understanding the mechanisms underlying the robust colonization and beneficial effects of *B. infantis* in the infant GI tract.

As we embark on a new era in nutrition of pediatric populations, this narrative review seeks to examine the existing understanding regarding *B. infantis*, encompassing both preclinical insights and clinical evidence. Through a narrative disceptation of the current literature and by critically evaluating the efficacy and potential mechanisms of action of *B. infantis* supplementation, we aim to elucidate its role in promoting optimal gut microbiome development and supporting the health of the pediatric population.

## 2. Framing the Context: The Current Understanding of the Field

### 2.1. The Unique Relationship between B. infantis and Human Milk Oligosaccharides (HMOs)

**Figure 1:**
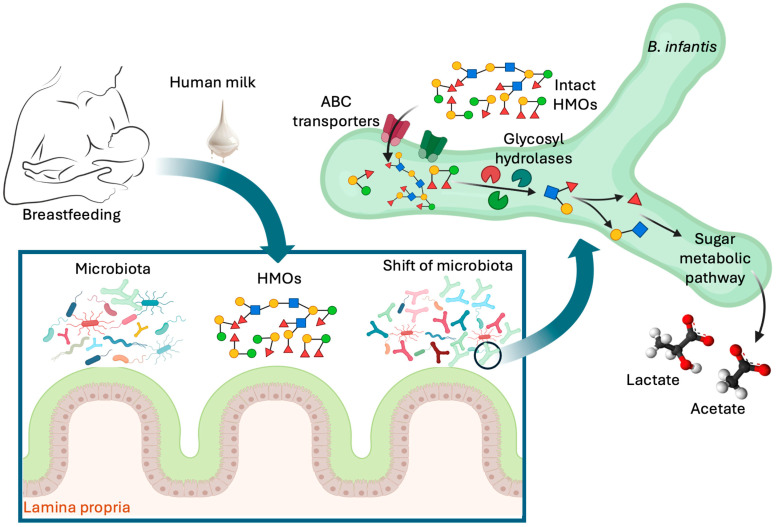
Role of *B. infantis* in utilizing HMOs for infant gut health.

Human milk is a rich source of human milk oligosaccharides (HMOs), a group of complex carbohydrates that the infant’s digestive system and most bacteria in the infant GI tract cannot break down [17]. This is due to the lack of the enzymes necessary to access and break down complex HMOs. However, *B. infantis* has evolved a remarkable ability to utilize these HMOs efficiently [17]. This symbiotic relationship between *B. infantis* and HMOs is critical for the establishment of a healthy GI microbiota in infants. HMOs serve as the primary energy source for *B. infantis* and, unlike other gut bacteria, *B. infantis* can metabolize the entire spectrum of these complex carbohydrates [17]. Researchers highlighted that *B. infantis* exclusively relies on HMOs for energy, a trait that provides it a significant competitive advantage in the gut of BF infants (see Figure 1) [17].

During breastfeeding, human milk oligosaccharides (HMOs) consumed by the infant specifically promote the growth of *Bifidobacterium longum* subsp. *infantis* (*B. infantis*) in the lower gut. This bacterium is uniquely equipped with specialized transport systems, including ATP-binding cassette (ABC) transporters, allowing it to efficiently bind, internalize, and metabolize HMOs. Inside the bacterial cell, HMOs are enzymatically broken down into simpler sugars by glycosidases, which are then used in central metabolic pathways to produce energy, biomass, and beneficial short-chain fatty acids (SCFAs). The dominance of *B. infantis* in the gut contributes significantly to the healthy development of breastfed infants.

Sequencing the genome of the original strain, *B. infantis* ATCC 15697, revealed an extensive array of genes specialized in the breakdown of complex carbohydrates [18]. This process involves several key steps. *B. infantis* possesses specific transport systems that enable it to bind and internalize HMOs from the intestinal lumen. These systems include solute-binding proteins and ATP-binding cassette (ABC) transporters, which are highly adapted to recognize and import a wide range of HMO structures. Once inside the bacterial cell, HMOs are subjected to enzymatic degradation. *B. infantis* produces a variety of glycosidases (such as α-sialidases, α-fucosidases, β-hexosaminadases and β-galactosidases, and α-sialidases) [18,19,20] that cleave the complex sugar structures of HMOs into simpler monosaccharides that are then metabolized through central metabolic pathways to produce energy and biomass. This process involves glycolysis and other catabolic routes that efficiently convert the monosaccharides into metabolites that support bacterial growth and function. Significantly, the genes responsible for the use of HMOs are preserved across all strains of *B. infantis* [21]. By effectively utilizing HMOs, *B. infantis* outcompetes other microbial species that are less efficient in HMO metabolism. This competitive advantage facilitates the establishment of *B. infantis* as a dominant member of the child’s GI microbiome, promoting a beneficial microbial environment. This specialization is evident in in vitro investigations showing that *B. infantis* can achieve cell densities three times higher than other species of bifidobacteria, such as *B. bifidum, B. adolescentis, B. longum* subsp. *longum*, and *B. breve*, when grown on HMOs as the sole carbon source [22,23]. The high efficiency in HMO utilization by *B. infantis* allows it to outcompete other microbes, thereby dominating the GI microbiota of BF infants. Finally, *B. infantis* carbohydrate carrier genes also play a role in defending against *Escherichia coli* O157:H7 [24].

### 2.2. Metabolic and Immunological Benefits of B. infantis

The metabolic activities of *B. infantis* produce several beneficial compounds that play vital roles in maintaining GI health and supporting the immune system. One of the key metabolic products are short-chain fatty acids (SCFAs), including lactate and acetate, that provide a crucial part in nutritional support and the development of the GI and immune system. Both acetate and lactate help lower the GI pH, creating an environment less conducive to the growth of harmful microorganisms [11]. Acetate, in particular, has multiple beneficial effects. It strengthens the GI barrier, reducing the permeability to harmful substances, supporting immune system maturation, providing essential substances to GI cells, and facilitating reciprocal feeding relationships that support the entire gut microbiome during early life [25,26,27].

Notably, Henrick et al. observed a gradual increase in the pH of stools in BF infants that have shifted from 5.0 to 6.5 over the past century, a trend linked to a decline in specialized bifidobacteria and a potential rise in the risk of microbiome dysbiosis [11]. In mouse studies, acetate has been found to stimulate GI antibody immunoglobulin (Ig)A production through the G-protein coupled receptor GPR43 [28]. Butyrate-producing bacteria in the gut, including *Faecalibacterium prausnitzii*, use acetate to synthesize butyrate, which serves as an energy source for gut cells and is essential for preserving GI barrier integrity, immune function, and brain health [29]. Consequently, HMOs foster the proliferation of bifidobacteria, which, through a process of cross-feeding, enhance the synthesis of butyrate, an important energy substrate for colon cells [30].

Beyond their GI impact, the SCFAs generated by *B. infantis* can permeate into the bloodstream, influencing various tissues such as the brain, lungs, liver and adipose tissue, thereby contributing to overall metabolic health [30]. Acetate is capable of crossing the blood–brain barrier: it can influence appetite regulation by acting on the hypothalamus, promoting feelings of satiety [31]. Lactate, on the other hand, can cross the blood–brain barrier, act as an energy source for the brain, and act as a neuromodulator [32].

Preclinical studies have shown that *B. infantis* enhances the integrity of the GI barrier by producing metabolites like indole-3-lactic acid (ILA), a tryptophan metabolite [33]. This metabolite helps to prevent the infiltration of harmful substances by maintaining the integrity of the tight junctions; moreover, it inhibits the expression of the interleukin (IL)-8, an inflammatory cytokine, in immature GI epithelial cells [26,34].

The SCFAs and other metabolites produced by *B. infantis* during HMO digestion exert various anti-inflammatory, antiviral, and cell developmental impacts on both mature and immature GI epithelial cells. They may provide protection against excessive GI inflammation, which is a common issue in premature infants, and they may help in maturing the immune response, which is crucial for protecting these infants from conditions such as NEC. *B. infantis* also generates compounds that support the development of the innate immune system. For instance, its metabolic by-products can inhibit the activation of pro-inflammatory pathways in the intestine, thus reducing the risk of chronic inflammation and associated diseases.

Besides the ability in fermenting carbohydrates, bifidobacteria are crucial for their interaction with the host’s immune system, affecting both adaptive and innate immune responses. Research has suggested that these bacteria might improve vaccine efficacy by boosting immunologic memory [10,35]. For instance, the link between fecal concentrations of bifidobacteria in healthy infants at the moment of vaccination and the antibody and T-cell reactions were examined in a prospective observational research [35]. Children who received vaccinations for hepatitis B virus (HBV), tetanus toxoid (TT), oral polio virus, and Bacillus Calmette–Guérin (BCG) were monitored with measurements of bifidobacteria levels taken at various points. The results indicated that infants with higher levels of bifidobacteria early in life exhibited more robust CD4 T-cell answers to TT, BCG, and HBV, along with higher polio-specific IgA in stool and TT-specific IgG in plasma after two years. The findings have been particularly significant for *B. infantis*, and this highlights the essential role of bifidobacteria in supporting vaccine and early immune responses.

*B. infantis* also plays an immunoregulatory role by enhancing the synthesis of IL-10, an anti-inflammatory cytokine, by T regulatory cells (T-regs) [36]. The modulation of specific immune cells and pathways by *B. infantis* has been demonstrated in both humans and animals, although the exact working vary among strains and may result in either pro-inflammatory or anti-inflammatory actions. In particular, *B. infantis* CCUG52486 has been shown to enhance the ratio of IL-10 to IL-12 n mononuclear cells from peripheral blood, suggesting potential benefits in controlling inflammatory processes. Maintaining the distribution of the categories of T-cells is crucial for the adaptive immune system’s homeostasis [2,37]. This acknowledges its importance in fostering a healthy microbiome and immune system, and potentially in preventing chronic diseases [38].

### 2.3. Early Host Adaptation: The Role of B. infantis in Shaping GI Microbiota

As previously discussed, the establishment of a GI microbiome predominated by *Bifidobacterium* spp. is critical for the healthy development of an infant’s GI and immune system The early colonization pattern of the GI microbiome profoundly influences both infant health and the individual’s long-term wellness [39,40,41]. Several factors strongly influence the composition of these early microbial populations [42] such as interruptions in vertical transmission (like delivery mode and the use of antibiotics during labor) [43,44], as well as disruptions in horizontal transmission (primarily influenced by feeding methods) [44,45]. Vaginal delivery results in greater prevalence of bifidobacteria in neonates compared with those born via cesarean section [46,47]; nevertheless, differences in the GI microbiota of newborns born via cesarean section and those delivered vaginally only become evident by the fifth day of life. Moreover, disparities in bifidobacteria levels between infants delivered vaginally versus by cesarean decrease by the time they reach 30 days of age [48], emphasizing the initial month after birth as crucial for establishing colonization. In addition, the abundance of *Bifidobacterium* spp. may be influenced by factors such as diet, antibiotic use, and puberty [4,49,50,51,52]. In BF infants, *B. infantis* becomes a dominant player, largely due to its ability to metabolize HMOs more effectively than other bacteria. This dominance is not merely a matter of numerical superiority but has profound implications on early childhood health and the individual’s future wellness. Conversely, infant formula (IF)-fed infants predominantly harbor species like *B. pseudocatenulatum* and *B. adolescentis*, which are typically more prevalent in the adult gut microbiota [53].

Regardless of the feeding method, the presence of *B. infantis* in the GI tract of infants is declining [54,55]. In some instances, *B. infantis* has not been detected at all during the initial six months of life [56]. Emerging studies indicate that the presence of *B. infantis* could rely on horizontal transmission among BF newborns, as this bacterium might not be passed from mother to child through vertical transmission. This was hypothesized because of its presence in the infant GI microbiome by two months of age [45]. Thus, this *Bifidobacterium* may face risks if breastfeeding ceases, as it requires contact between BF newborns and their mothers to survive.

In industrialized nations, as both breastfeeding rates and interactions among BF infants have declined, these bifidobacteria spp. are now rarely observed, even in infants who have not been exposed to antibiotics [57]. The absence of *B. infantis* is also believed to be due to its scarcity in the adult GI tract and its disappearance from the mother’s GI microbiome, potentially due to the use of antibiotics in mothers and various practices that alter the microbiome [57]. Therefore, if *B. infantis* is absent from the mother’s GI tract, the infant does not acquire this important bacterium during birth, which is a key method for establishing their GI microbiota.

These studies point to a worrying trend: *B. infantis* is at risk of extinction [57,58], especially in regions with traditionally low breastfeeding rates and shorter breastfeeding durations [11]. However, when infants are supplemented with *B. infantis*, their gut microbiotas exhibit a dramatic shift.

In a study conducted by Underwood et al. [59], premature infants (*n* = 12, five weeks old) participated in two phases. During phase one, infants receiving IF were randomly assigned to be administered either *B. infantis* ATCC 15697 (4 billion colony forming units (CFU) every twelve hours) or *B. animalis* subsp. *lactis*, with dosages escalating over the course of five weeks. In the second phase, nine premature infants fed with human milk were given each bifidobacteria strain for two weeks, with a one-week washout period in between. The fecal bifidobacteria levels were notably higher in the ATCC 15697 group, while Proteobacteria levels were significantly reduced during phase two compared with the *B. animalis* subsp. *lactis* group. The authors concluded that ATCC 15697 more effectively colonized premature infants than *B. animalis* subsp. *lactis* [59]. Additionally, the study showed that the combination of *B. infantis* and human milk proved to be the most successful in bringing the fecal microbiota to a balanced state, highlighting *B. infantis’* specialized capacity to break down HMOs.

Studies have shown that exclusively BF infants receiving *B. infantis* EVC001 have a GI microbiome where *Bifidobacterium* spp. constitute up to 80% of the total microbiota, with *B. infantis* alone accounting for 90% of the Bifidobacterium population [16]. Additionally, stool HMO levels were notably lower in the EVC001 group, indicating a rise in bifidobacteria metabolism. Additionally, lactate and acetate levels were elevated in the EVC001 group, altering the intestinal environment to inhibit pH-sensitive pathogen growth. Infants colonized by EVC001 had a lower fecal pH (5.15) and reduced fecal endotoxins compared with non-colonized infants, reflecting decreased Proteobacteria and Bacteroidetes levels [60]. This near-monoculture of beneficial bacteria significantly reduces the presence of potential pathogens, which make up less than 10% of the gut community in these infants. In contrast, exclusively BF infants not supplemented with *B. infantis* have a reduced abundance of bifidobacteria and an increased prevalence of pathogenic bacteria. Finally, infants treated with EVC001 showed significantly reduced calprotectin and pro-inflammatory cytokines in fecal samples, indicating reduced enteric inflammation [61].

In another multicenter double-blind randomized controlled trial (RCT), full-term infants were randomly assigned to two groups. In the first group, 97 infants received a standard IF. In the second group, 93 were given an IF supplemented with *B. infantis* CECT 7210 at 10 million CFU per 100 mL of IF. This regimen was followed over a 12-week feeding period [62]. By the end of the study, total stool bifidobacteria counts were comparable, and *B. infantis* levels were notably elevated in the group receiving CECT 7210.

Healthy term infants aged 3 to 12 months (*n* = 208) were enrolled in a study where they were administered *B. infantis* R0033 at a dose of 3 billion CFU per day for a duration of eight weeks. [63]. They showed a marked reduction in fecal genera such as *Klebsiella, Enterococcus, Collinsella*, *and Blautia,* along with a rise in the ratio of IL-10 to IL-12, indicating potential anti-inflammatory and anti-pathogenic effects [64]. The R0033 anti-inflammatory activity may be related to the synthesis of ILA, although ILA levels were not measured in this specific study.

In another RCT, infants supplemented with *B. infantis* M-63 from their first week until three months old exhibited improved gut microbiota composition increasing bifidobacteria levels, GI function, and immune parameters compared with those receiving a placebo [65]. M-63 effectively utilized HMOs and reduced pH in stools, elevated IgA levels in the blood and fecal acetic acid, and improved stool consistency without adverse effects [66]. M-63 also shows potential in promoting healthy GI microbiota in preterm and low-birth-weight infants, who are at risk of dysbiosis due to delayed colonization by beneficial bacteria and frequent antibiotic use [66]. A study involving low-birth-weight infants found that a triple-strain probiotic mixture, including *B. infantis* M-63, led to a faster establishment of a microbiota dominated by bifidobacteria and reduced levels of *Enterobacteriaceae* compared with single-strain supplementation [67]. Another study with extremely preterm infants demonstrated that probiotics containing M-63 used in both single-strain and multistrain formulations improved gut microbiota composition and reduced dysbiosis, facilitating faster feeding milestones [68,69].

This highlights the importance of strain-specific probiotics in supporting a healthy GI environment in infants.

## 3. The Therapeutic Potential of *B. infantis* in Infants and Children: Insights from Preclinical and Clinical Research

Over the course of life, numerous health issues have been connected to disruptions in the GI microbiota balance. In premature infants, improper colonization of the GI tract is regarded as a contributing factor to NEC [70,71]. An imbalance in the GI microbiome has been correlated with disorders affecting the cardiovascular, digestive, metabolic, and nervous systems, as well as with autoimmune conditions and allergies at various life stages [72,73]. In detail, the reduction in *Bifidobacterium* in the GI microbiome of infants and the resulting imbalance of microbial communities, characterized by an increase in harmful microorganisms, has been proposed as a potential factor to the rising occurrence of autoimmune conditions in developed countries [74]. Bifidobacteria are generally considered beneficial for host health, acting directly and indirectly to prevent infections, boost immunity, inhibit pathogenic bacteria, and manage inflammatory diseases [75]. These effects extend beyond the GI tract, influencing overall host homeostasis.

While probiotics are extensively studied in adults, their most significant impact appears to be in infants and children. *B. infantis*, in particular, is frequently used in research and clinical trials, showing promise, either alone or in combination with other probiotics, in treating conditions like NEC in premature infants, childhood diarrhea, functional GI disorders (FGID), IBD, and in preventing allergies. Recent key research findings that illustrate the benefits of *B. infantis* in the infant and child population are summarized in this section.

### 3.1. Gastrointestinal Effects

In recent years, research has progressively demonstrated the efficacy of *B. infantis* in reducing various GI diseases and disorders, fostering bifidobacteria colonization [67,76,77] and promoting overall GI health [78,79,80].

In scenarios where natural colonization is disrupted, supplementing with *B. infantis* can provide a “reseeding” effect, restoring the gut microbiota to support optimal health outcomes. In 2023, the European Society for Pediatric Gastroenterology, Hepatology and Nutrition (ESPGHAN) released recommendations on using probiotics to prevent necrotizing enterocolitis (NEC) for the management of mildly active ulcerative colitis and FGID [81]. They were largely derived from strain-specific network meta-analysis and pair-wise systematic reviews and they highlighted that specific probiotic strains administered orally, including *B. infantis*, are effective for the management of some pathologies, such as in preventing NEC in premature infants. Despite evidence supporting the administration of targeted probiotics in selected medical scenarios, additional research is often required to validate their effects and determine the appropriate type, dose, and timing.

The main research involving *B. infantis* are listed in Table 1.

#### 3.1.1. Necrotizing Enterocolitis and Late-Onset Sepsis

NEC affects an estimated 5–12% of preterm infants weighing less than 1500 g at delivery, resulting in mortality rates reaching 20–30% [98,99]. Numerous risk factors have been identified across prenatal, perinatal, and neonatal periods [100], such as aberrant colonization of the infant gut by microorganisms and GI immaturity [101]. Before NEC develops in preterm infants, the GI microbiota is often marked by a reduction in the diversity of microbes [98,102,103], a higher presence of Proteobacteria, coupled with diminished levels of Bacteroidetes and Firmicutes, and a significant reduction in commensal bacteria such as bifidobacteria [104,105].

Preterm infants exhibit an atypical pattern of GI microbiome colonization influenced by several factors, which elevate their risk for GI and atopic diseases, particularly NEC [106]. Several studies in animal models have been conducted to understand the effects of probiotics in preventing NEC. For example, Lu et al. reviewed various NEC animal models, where strains of *B. infantis* were tested for their efficacy [107]. One complex study in a preterm murine model revealed that a probiotic combination with *B. infantis* was the one that most effectively prevented NEC. This combination, including *B. infatis* and *B. bifidum*, reduced *Escherichia coli* and *Klebsiella* colony counts in fecal samples and decreased mortality rates among the animals [108]. Additional rodent studies revealed that *B. longum* could reduce inflammatory markers including iNOS, Cxcl1, and IL-23, thus preventing NEC, and inhibit the proliferation of clostridial species [27,109]. Transitioning to human studies, several meta-analyses of over 25 RCTs have demonstrated that probiotic supplementation significantly decreases the incidence of NEC in preterm infants. This reduction is attributed to the promotion of beneficial microorganism colonization, the enhancement of intestinal barrier function, and the development of the immune system [110,111,112,113,114]. For instance, several clinical trials with *B. infantis* provide evidence supporting these benefits (refer to Table 1 for additional trial details). Ishizeki et al. compared a single-species probiotic (*B. breve* M-16V) with a multispecies probiotic (*B. breve* M-16V, *B. longum* ssp. *longum* BB536 and *B. infantis* M-63) in preterm infants, finding an elevation in bifidobacteria and a reduction in *Enterobacteriaceae* and *Clostridium* in those receiving the multispecies preparation [67]. Underwood et al. assessed the effects of *B. animalis* ssp. *lactis* UCDavis 316 and *B. infantis* ATCC15697 on the GI microbiome of preterm infants receiving either breast milk or formula. They found *B. infantis* ATCC15697 to be a superior colonizer and modulator, particularly enhancing bifidobacteria levels and microbiota diversity in IF-fed infants [59]. These findings underscore the importance of selecting the appropriate probiotic strains for effectively modulating the GI microbiota of neonates born prematurely.

In the ProPrems trial, a prospective multicenter double-blind RCT, Jacobs et al. investigated the effect of a mixture of bacterial species on the incidence of late-onset sepsis in premature infants born prior to 32 weeks of gestation [87]. Infants were randomly assigned to receive a daily probiotic combination (*n* = 548; *B. infantis* BB02, *B. lactis* BB12, *Streptococcus thermophilus* TH4; 1 billion CFU per day) or a placebo (*n* = 551). The probiotic combination significantly reduced the occurrence of NEC of Bell stage 2 or higher, although no significant differences were observed in confirmed late-onset sepsis or overall mortality between the groups. A subsequent study found that this probiotic formulation elevated levels of *Bifidobacterium* (*p* < 0.001) and decreased concentrations of *Enterococcus* (*p* = 0.02) in the GI microbiome of very premature infants, suggesting a protective role of Bifidobacterium in relation to NEC [115].

Despite the widespread recognition of probiotics in reducing NEC, the majority of studies report minimal effects on late-onset sepsis [83,87], except for findings from Lin et al. [84] and Samanta et al. [85]. Many investigations also note a notable decrease in death rate, with Jacobs et al. [87] being an exception. Over 5000 premature infants have participated in RCTs involving probiotic treatments, with no associated sepsis reported [116]. However, current research has identified five cases of bacteremia related to probiotics in infants born prematurely receiving *B. infantis* formulations. Among these cases, two infants were asymptomatic and did not need antibiotic intervention, while a third required antibiotics, and two more developed NEC but survived after surgical and antibiotic treatment [117,118]. Despite these sporadic instances, the regular use of probiotics with very low-birth-weight (VLBW) infants in neonatal units has persisted in growing with few adverse effects reported, reinforcing the safety of probiotic supplementation [119,120]. Most RCTs and meta-analyses support the use of *B. longum* strains for NEC prevention or treatment.

In particular, ESPGHAN has made conditional recommendations for the utilization of the mixture of *B. lactis* BB-12, *B. infantis* BB-02, and *Streptococcus thermophilus* TH-4 at a concentration of 3 to 3.5 × 10^8^ CFU for every strain, based on a low certainty of evidence [81]. Nevertheless, further clinical research is essential to identify the optimal probiotic agents and dosing regimens.

The FDA has recently raised concerns about the use of probiotic products in hospitalized preterm infants, highlighting potential safety and efficacy issues in this vulnerable population [121]. While some studies suggest that probiotics may reduce the risk of NEC, the FDA emphasizes that these products are not approved for therapeutic use in neonates and pose potential risks, including systemic infections. The agency urges caution and calls for further clinical research to evaluate the safe and suitable application of probiotics in these delicate patients [121].

#### 3.1.2. Functional Gastrointestinal Disorders

Infant colic (IC), characterized by prolonged crying and abdominal discomfort of unknown origin, is a common issue potentially linked to variations in the GI microbiome [122]. Researchers suggest that infants with IC exhibit lower levels of beneficial GI bacteria including bifidobacteria and lactobacilli. In an RCT, the effects of probiotic supplementation with *B. infantis* M-63 (10 million CFU/g) and *Lactobacillus rhamnosus* (now *Lacticaseibacillus rhamnosus*, *L. rhamnosus*) LCS-742 (10 million CFU/g) in an α-lactalbumin-enriched IF were examined for IC incidence, nutritional adequacy, and GI tolerance [78]. The study involved 66 infants receiving formula who were between 3 weeks and 3 months old, and, over a one-month feeding period, infants receiving *B. infantis* M63 experienced fewer feeding-related GI symptoms, despite the absence of a notable difference in crying duration among the groups [78]. These findings suggest that α-lactalbumin-enriched IF supplemented with probiotics like *B. infantis* M-63 supports appropriate growth and enhances GI comfort in infants suffering from IC. The identical probiotic combination was evaluated in a separate RCT involving 97 infants born at term who received an IF enriched with *B. infantis* M-63 and *L. rhamnosus* LCS-742, each at a concentration of 140 million CFU/g, for a duration of six months [93]. Following one month, infants fed with probiotics showed reduced episodes of agitation or crying and demonstrated calmer behavior (*p* = 0.03). By six months, a lower occurrence of atopic dermatitis was observed (*p* < 0.05). These studies collectively indicate potential benefits, including improved tolerance and protection against atopic dermatitis in infants with IC.

Another probiotic mix consisting of *B. infantis* M63 (1 billion CFU per day), *B. longum* BB536 (3 billion CFU per day), and *B. breve* M16 (1 billion CFU per day) was investigated in various RCTs demonstrating potential in alleviating GI conditions such as IBS [79] and chronic functional constipation [80]. These conditions, which can persist into adolescence for many children [123], may benefit from probiotics as adjunct therapies, potentially mitigating polyethylene glycol (PEG)-related gut microbiota imbalances [124]. For instance, children aged 4–12 years (*n* = 55) with functional constipation received PEG alone or in combination with the probiotic combination administered for a duration of eight weeks [125]. While the probiotic mixture was deemed beneficial, no significant difference in effectiveness was observed between the groups. In another crossover RCT, 48 children aged 8–16 years with IBS and 25 with functional dyspepsia were administered the probiotic combination or placebo for 6 weeks [79]. Probiotic treatment alleviated abdominal pain in patients with IBS; however, it did not have the same effect on those suffering from functional dyspepsia. Furthermore, following treatment, 48% of pediatric IBS patients experienced an enhanced quality of life, compared with 17% of those receiving the placebo (*p* = 0.001). Nevertheless, the study’s two-week washout period may not have been sufficient to eliminate the potential for a “carryover” effect between treatments. While these trials utilized a probiotic mixture that included *B. infantis* M63, the specific advantages are not exclusively attributable to *B. infantis* but align with the known mechanisms associated with this strain.

#### 3.1.3. Infectious Gastroenteritis

Infectious gastroenteritis is a major global health issue, causing over two billion diarrheal episodes annually and significant mortality in children under five in developing nations [126]. While oral rehydration is standard for managing dehydration, it does not decrease diarrhea incidence or duration, nor does it expedite GI normalization. Probiotics have been suggested as an adjunctive treatment, showing particular benefits for pediatric populations [127]. Enteric bacterial and parasitic infections are more common than viral infections in developing countries, with peaks typically occurring during the summer seasons. A leading cause of bacterial gastroenteritis worldwide, particularly risky for infants and immunocompromised elderly, are *Salmonella* spp. [126]. In vivo studies have shown that *B. infantis* 35624 can reduce inflammatory responses by decreasing IFN-γ and IgG2 levels in gnotobiotic mouse models challenged with *Salmonella Typhimurium* [128]. Additionally, Symonds et al. [129] observed that treatment with *B. infantis* 35624 attenuated Salmonella-induced damage to brush border enzyme activity and reduced weight loss in mice.

Acute diarrhea is predominantly caused by viral infections worldwide, impacting both developed and developing regions. Despite being perceived as a manageable childhood illness, rotavirus infections lead to numerous hospitalizations and deaths annually among children under five years of age [130]. Probiotics have been investigated as treatments and/or preventatives for rotavirus infections. These studies, conducted with animal models and through clinical trials, have shown varied outcomes depending on the specific probiotic strains used, primarily *Limosilactobacillus* spp. [131,132]. *B. infantis* has also been tested and shown promising results. In vitro studies using MA-104 and HT-29 cell lines have shown that *B. infantis* IM-1 can inhibit rotavirus replication (up to 36.05%) and protect cells from rotavirus infection (up to 48.50%). In murine models, *B. infantis* CECT 7210 (also known as *B. infantis* IM1), extracted from the stool of a BF newborn, demonstrated initial protection against rotavirus by reducing viral counts in stools and bolstering mucosal defenses, which is reflected in increased IgA concentrations [132,133].

In a multicenter double-blind RCT, Escribano et al. evaluated the effectiveness of this strain to diminish the incidence of diarrhea in healthy full-term infants over a period of twelve weeks [62]. The median number of diarrhea events per infant during the study was 0.05 ± 0.28 in the *B. infantis* IM1 group compared with 0.29 ± 1.07 in the control group (*p* = 0.059), achieving statistical significance by the eighth week (*p* = 0.047). Nevertheless, the overall incidence of diarrhea was low, likely because the participants were very young (under 3 months) and they may still have had maternal antibodies that offered protection.

Another commercial probiotic formulation containing three bacterial strains (*B. bifidum* R0071, *B. infantis* R0033, and *Lactobacillus helveticus* R0052) was tested in an RCT involving children aged one to five years. The results indicated that it was effective in decreasing both the duration and severity of diarrhea caused by rotavirus [134].

In a 2024 RCT conducted in the Kurdistan Region, Abdulah et al. investigated a probiotic mixture of *B. infantis*, *L. paracasei*, and *L. rhamnosus* with a dose of 10 × 10^6^ CFU per day and zinc supplementation administered for 7 days in infants and children (*n* = 101) with acute infectious diarrhea. While the probiotics plus zinc group did not significantly impact disease severity compared with the probiotics-only group after two weeks, it did demonstrate faster recovery times (1.34 days vs. 2.00 days, *p* < 0.001). Both groups experienced significant reductions in dehydration severity and disease scores, as well as decreased incidence of mild gastroenteritis from baseline to 2 weeks. These findings suggest that while probiotics plus zinc did not alter disease severity outcomes significantly, it did enhance recovery rates in children with acute gastroenteritis [96].

Positive outcomes have been achieved in treating and preventing acute diarrhea using *B. infantis*. Nonetheless, additional research is required to ascertain the optimal bacterial dosage, especially when several species are incorporated into the supplement.

#### 3.1.4. Inflammatory Bowel Disease (IBD)

Ulcerative colitis (UC) is characterized by recurrent inflammation of the colon and rectum, presenting symptoms including bloody diarrhea, tenesmus, fecal urgency, and abdominal pain [135]. Together with Crohn’s disease (CD), an inflammatory condition that may affect any section of the intestine, UC falls under the category of IBD.

In a Cochrane review from 2020 which examined the impact of probiotics on inducing remission, it was found that there is limited evidence suggesting probiotics might induce clinical remission in individuals with active UC compared with a placebo [136]. Nonetheless, the review did not identify specific strains, and it only included two randomized controlled trials involving pediatric patients.

In one of these trials, Miele et al. explored the use of a multistrain probiotic mixture of eight strains (including *L. delbrueckii* subsp. *bulgaricus* DSM 24734, *L. acidophilus* DSM 24735, *L. plantarum* DSM 24730, *L. paracasei* subsp. *paracasei* DSM 24733, *B. breve* DSM 24732, *B. infantis* DSM 24737, *B. longum* subsp. *longum* DSM 24736, and *Streptococcus salivarius* subsp. *thermophilus* DSM 24731), alongside standard IBD therapy, for treating newly diagnosed UC in children [97]. The study randomized 29 children to receive either the probiotic mix incorporated with 5-aminosalicylic acid (5-ASA) and steroids or a placebo. The probiotic group had a significantly higher remission rate (92.8% vs. 26.7%, respectively, *p* < 0.001) and a lower relapse rate over one year (21.4% vs. 73.3%, *p* = 0.014). The authors attributed the efficacy of the formulation to the elevated bacterial concentration of 3 × 10^11^ cells/g and the diverse range of strains. However, the trial’s small sample size (*n* = 29) indicates a need for confirmatory studies with larger patient populations.

However, according to ESPGHAN’s methodology, a minimum of two RCTs with the same strains is necessary to make a recommendation [81]. Therefore, no recommendation was made regarding the application of probiotics in managing children with UC in the 2023 document.

In mouse models with dextran sodium sulfate (DSS)-induced colitis, *B. infantis* B8762 (VB8762) and its non-viable probiotic cells, known as postbiotics, decreased body weight loss, reduced mortality, had lower disease activity index scores, and improved histology scores. These findings underscore the potential of postbiotics in addressing the challenges associated with the use of live probiotics, particularly in vulnerable populations and clinical settings where safety is paramount [137].

#### 3.1.5. Severe Acute Malnutrition

Observational studies have highlighted a link between the decreased prevalence of *B. infantis* and severe acute malnutrition (SAM) in children from Bangladesh. This indicates that *B. infantis* may be essential in the nutritional status of neonates [138]. In healthy infants from Bangladesh, around 75% of the bifidobacterial strains identified in stool samples during the first year of life are *B. infantis*, owing to its swift colonization during the initial month. Conversely, infants suffering from SAM exhibit significantly lower levels of *B. infantis*—by 2 to 3 orders of magnitude—compared with their healthy counterparts. Instead, their gut microbiota is predominantly composed of potential pathogens such as *Escherichia, Shigella, Klebsiella*, and *Streptococcus* spp. Other studies reinforce the link between malnutrition and hindered development of the GI microbiome, which attribute potential causes to factors like dysbiosis-induced diarrhea and exposure to environmental pathogens [139,140,141]. The supplementation of probiotics or synbiotics is considered a possible clinical strategy to enhance health and nutrition outcomes in malnourished children [142]. A clinical trial involving Bangladeshi infants with SAM demonstrated that supplementing with *B. infantis*, with or without lacto-N-neotetraose, significantly boosted the level of *B. infantis* and facilitated weight gain more effectively than the placebo group. However, the levels of *B. infantis* in the supplemented group remained 1 to 2 orders of magnitude lower compared with those in healthy infants, likely due to the low breastfeeding rates among the SAM group. Yet, the partial recovery of the GI microbiome and the corresponding growth improvements underscore the significance of *B. infantis* and its potential role in addressing global child undernutrition. Developing preclinical investigations also suggest that *B. infantis* has significant implications for the skeletal and neural systems via the gut–bone and gut–brain axes [143,144]. Supplementation with *B. infantis* has been shown to enhance overall skeletal length and stimulate the function of bone-remodeling cells by activating the PI3K/AKT pathway through the growth hormone/insulin-like growth factor-1 axis in growing mice [66].

### 3.2. Extraintestinal Effects

The microbiota–gut–brain axis refers to the bidirectional communication channels linking the GI microbiota and its metabolites with the enteric, central, and autonomic nervous systems, as well as the hypothalamic–pituitary–adrenal axis. Specific components of the GI microbiota, such as *B. longum*, have been demonstrated to synthesize neurochemicals like gamma-aminobutyric acid (GABA), a key inhibitory neurotransmitter, or participate in regulating host serotonin biosynthesis. Additionally, SCFAs produced by *B. infantis* are implicated in the microbiota–gut–brain axis, potentially influencing mood, cognition, and the etiology of brain disorders either directly or indirectly.

In this section, we will thoroughly analyze how *B. infantis* influences various aspects of health beyond the gut, considering findings from multiple studies (see Table 2).

#### 3.2.1. Autism Spectrum Disorders

In 2012, it was estimated that 1 in 160 children globally had a pervasive developmental disorder, including autism spectrum disorder (ASD) [149] with later reports indicating a rising prevalence globally [150]. Comorbidities often include GI issues such as diarrhea, constipation, and abdominal pain [151]. Wang et al. conducted an RCT to investigate the effects of synbiotic formulation on ASD symptoms, gut microbiota composition, neurotransmitter levels, and SCFA concentrations [146]. In this study, the synbiotic included four bacterial strains (*B. infantis* Bi-26, *L. rhamnosus* HN001, *B. lactis* BL-04, *L. paracasei* LPC-37) along with the prebiotic fructo-oligosaccharides (FOSs). Prior to the intervention, differences were observed in fecal microbiota composition, SCFA production, and plasma neurotransmitter levels between children diagnosed with ASD and typically developing children [152]. Specifically, children with ASD had lower levels of beneficial bacteria such as *B. longum* and Bifidobacteriales, and higher levels of *Ruminococcus* and *Clostridium*. SCFAs like butyrate, propionate, and acetic acid were also significantly reduced in children with ASD. Additionally, these children exhibited elevated serotonin levels and reduced homovanillic acid, a marker of dopaminergic activity in the central nervous system. During the 108-day intervention period, the synbiotic led to significant increases in beneficial bacteria like *B. longum* (*p* < 0.001 at day 108) and decreased levels of suspected pathogens such as *Clostridium* spp. (*p* < 0.05 at day 108) compared with the placebo group. It also resulted in elevated SCFA levels and increased homovanillic acid, while reducing serotonin levels—effects not observed in the placebo group. Although the synbiotic did not affect other neurotransmitters and metabolites such as GABA, acetylcholine, histidine, arginine, histamine, glutamine, and glutamic acid, it did improve GI symptoms and reduced the severity of ASD symptoms, as measured by the Autism Treatment Evaluation Checklist (ATEC) [146].

In a separate crossover study, children with ASD who experienced chronic constipation, diarrhea, or IBS (*n* = 11, aged 2 to 11 years) participated. These children received a combination of *B. infantis* UCD272 (20 billion CFU/day) and a prebiotic (bovine colostrum product) over five weeks, followed by a two-week washout period and then five weeks of the prebiotic alone [145]. The *B. infantis* UCD272 probiotic showed a tendency towards greater improvement in immune imbalances, aberrant behavior, and GI symptoms during the period when the prebiotic was used alone. However, the conclusions of the study were limited by the small number of participants involved.

#### 3.2.2. Respiratory Diseases and Allergies

Upper respiratory tract infections encompass viral or bacterial infections affecting the nose, pharynx, larynx, sinuses, and large airways, ranking among the top three diagnoses in outpatient settings [20]. In 2003, non-influenza-related viral respiratory infections in the United States were estimated to incur an annual economic burden exceeding $22 billion [153]. Seasonal allergies, such as hay fever or allergic rhinitis, occur during periods of high pollen counts, presenting symptoms like runny nose, watery eyes, coughing, and sneezing. Among allergic 2-year-old children, the GI microbiota typically shows lower levels of bifidobacteria, lactobacilli, and Bacteroides, and higher levels of aerobic microorganisms like *Staphylococcus aureus* compared with non-allergic peers. Therefore, interventions using beneficial bacteria have the potential to mitigate or prevent the severity of respiratory illnesses and seasonal allergies by influencing the gut microbiota, its functionality, and host immunity.

In a multicenter pilot study, children with a history of at least three episodes of common winter illnesses (ear, nose, and throat infections, respiratory tract infections, or GI illnesses) received a synbiotic formulation for 3 months to assess its efficacy in preventing these conditions [147]. The synbiotic comprised *B. infantis* R0033, *B. bifidum* R0071, *L. helveticus* R0052 (total of 3 billion CFU), and FOSs. Compared with the placebo, the synbiotic led to a 25% relative risk reduction in the incidence of common infectious diseases during the treatment period (*p* = 0.045) and reduced the number of school days missed (*p* = 0.043). Similar to other studies reviewed, the observed benefits cannot be solely attributed to R0033 alone. However, the reduction in infectious diseases, including GI illnesses, aligns with known mechanisms of *B. infantis*, such as inhibiting the growth of pH-sensitive pathogenic organisms.

In another RCT, children aged 2–6 years consuming *B. longum* BB536 for 10 months experienced alleviation of upper respiratory illness symptoms [154]. Specifically, compared with the placebo, the strain reduced the duration of sore throat by 46% (*p* = 0.018), runny nose by 15% (*p* = 0.087), and cough by 16% (*p* = 0.087). Analysis of the gut microbiota revealed an increase in the *Faecalibacterium* genus in the BB536 group over 10 months, a change not observed in the placebo group (*p* < 0.05).

A combination of *B. longum* BB536 with *B. infantis* M-63 and *B. breve* M-16V may effectively alleviate allergic conditions such as intermittent asthma and allergic rhinitis triggered by pollen and improved quality of life [148]. However, the small sample size (*n* = 40) in this trial underscores the need for larger trials to validate these findings.

Additionally, *B. infantis* M-63 can persist in the gut microbiota of infants with cow’s milk allergy (CMA), leading to beneficial changes in microbial composition and potentially supporting immune tolerance [77]. Infants with CMA typically show a dysbiotic gut microbiota, and *B. infantis* M-63 supplementation has been associated with increased levels of beneficial bacteria like *Akkermansia* spp. and *Ruminococcus* spp. [77].

## 4. Safety of *B. infantis*

Numerous clinical studies have thoroughly assessed the safety of *B. infantis*, including genomic, functional, and in vivo analyses. These assessments have consistently confirmed that *B. infantis* is non-toxigenic, non-pathogenic, non-resistant to antibiotics, non-hemolytic, and it does not carry plasmids [155,156,157,158,159].

These studies involved over 800 infants at various developmental stages, including healthy full-term infants [65], premature infants [67,68,116], infants with IC [78], allergies [77,93], and those recovering from GI surgeries [76].

Additionally, more than 160 children aged 4–17 years with GI issues [79,80] or allergies [148] were included in these studies. Conducted in multiple countries such as Italy, France, Japan, Australia, and other countries, these trials consistently reported that *B. infantis* is safe and well-tolerated, with no adverse effects observed in either healthy or medically challenged infants following consumption of this probiotic strain.

*B. infantis* has demonstrated significant benefits in promoting a healthy GI microbiota. When combined with other probiotic strains, *B. infantis* enhances bifidobacteria colonization and improves GI tolerance, thereby supporting general health during infancy. Moreover, *B. infantis,* alone or in combination with other bacterial species and prebiotics, has shown potential in alleviating symptoms associated with FGID and allergies and may mitigate the severity or prevent the onset of various diseases, including non-communicable diseases, from early life through adulthood.

In summary, clinical studies provide robust evidence supporting the safety and efficacy of *B. infantis*, making it suitable for promoting nutrition and health in infants and children across a wide range of conditions.

## 5. Discussion

**Figure 2:**
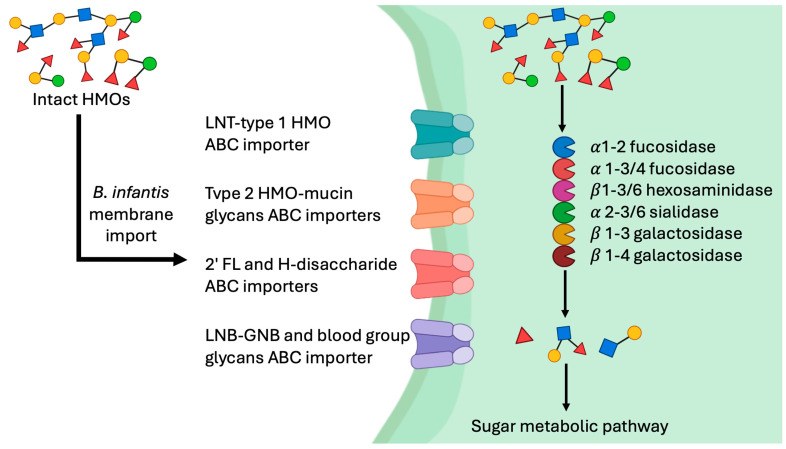
Strategies for HMOs utilization by *B. infantis.* The image illustrates how HMOs are imported in *B. infantis* through specific ABC transporters and then degraded by glycosyl hydrolases such as fucosidases, sialidases, and galactosidases. This breakdown releases sugars that fuel *B. infantis* growth, providing it a competitive advantage in the gut of breastfed infants.

The interaction between *Bifidobacterium* and nutrition is deeply rooted in the molecular capabilities of these bacteria to metabolize complex carbohydrates, particularly HMOs [160]. In breastfed infants, *B. infantis* is one of the key microbial species that thrive due to its specialized genetic machinery for HMO digestion [66]. This process is critical because most gut microbes are unable to utilize HMOs, giving *B. infantis* a competitive edge [160]. One of the most notable features of the *B. infantis* genome is the presence of a 43 kb gene cluster, known as HMO cluster I, which contains several genes involved in the import and metabolism of HMOs [161]. This cluster includes glycosyl hydrolases, including α-sialidases, α-fucosidases, and β-galactosidases, which break down HMOs into usable sugars, such as fucose, sialic acid, and glucose, and oligosaccharide transport proteins that enable the bacteria to import HMOs from the gut lumen into the cell (Figure 2). These sugars fuel the growth of the bacteria, allowing it to dominate the gut environment of breastfed infants [162].

The consumption of HMOs by *B. infantis* also leads to the production of SCFAs, including acetate and lactate. These SCFAs play an essential role in maintaining gut health, as they lower gut pH, inhibit the growth of pathogenic bacteria, and support the development of the gut epithelial barrier [162]. Molecularly, SCFAs interact with host G-protein-coupled receptors (such as GPR43) on immune cells, triggering anti-inflammatory pathways that are crucial for protecting the neonatal gut from excessive immune reactions. SCFAs also serve as energy sources for colonocytes, reinforcing the integrity of the gut lining [163].

Moreover, *B. infantis* engages in cross-feeding interactions with other gut microbes, further shaping the infant gut microbiome [164]. By breaking down HMOs into smaller by-products, *B. infantis* facilitates the growth of other beneficial bacteria that can utilize these simpler sugars [164]. This synergy contributes to the establishment of a healthy, well-balanced microbiota, which is essential for the infant’s immune system development and overall health [165]. The genetic and enzymatic adaptations of bifidobacteria to utilize HMOs have significant implications for infant health [166]. By selectively enriching bifidobacteria in the infant gut, breast milk supports the establishment of a microbiota that is optimized for nutrient absorption, pathogen defense, and immune system modulation [166]. The dominance of bifidobacteria in the gut of breastfed infants is associated with lower rates of GI infections, reduced inflammation, and improved immune function [167].

At the sub-molecular level, *Bifidobacterium* interacts with the host’s immune system through a series of complex mechanisms. One key process is the modulation of the host’s Tregs, which are critical for maintaining immune tolerance [168]. *Bifidobacterium* species, particularly *B. infantis*, have been shown to induce the production of the anti-inflammatory cytokine IL-10, which enhances the function of Tregs [36]. This cytokine helps regulate immune responses, reducing the risk of inflammation-driven diseases such as NEC in preterm infants [36].

Furthermore, *Bifidobacterium* produces metabolites such as ILA, which is derived from the fermentation of tryptophan [169]. ILA has been found to play a protective role in maintaining intestinal barrier function by promoting tight junction integrity. This ensures that harmful pathogens and toxins are kept out of the bloodstream, thus preventing systemic inflammation [169].

The protective effects of *Bifidobacterium* are not limited to local gut interactions. SCFAs, particularly acetate, can cross the blood–brain barrier, where they exert neuroprotective effects by acting as an energy source for neurons and modulating neuroinflammation [143]. This connection between the gut and the brain, commonly referred to as the gut–brain axis, suggests that *Bifidobacterium* may have broader implications for cognitive and neurological development in infants [143].

The co-evolution of bifidobacteria and human milk highlights the importance of understanding the molecular mechanisms that underlie this symbiotic relationship. Systems biology approaches, such as metabolomics and next-generation sequencing, are poised to shed light on the interactions between HMOs, the infant microbiome, and host health [106]. These insights could inform the development of novel nutritional interventions, including probiotics and prebiotics, that mimic the beneficial effects of HMOs in formula-fed infants or those with disrupted microbiota.

In addition to understanding the molecular linkages between bifidobacteria and nutrition, researchers are exploring innovative methods to enhance the delivery, the efficacy, and stability of probiotics like *B. infantis*. One promising approach is bacterial encapsulation, which involves coating probiotic bacteria with protective materials to shield them from harsh GI conditions, such as stomach acid and bile salts [170]. Encapsulation improves the viability of probiotics during transit through the digestive system, increasing the likelihood that they will reach the colon in sufficient numbers to exert their beneficial effects. Encapsulated probiotics also offer the advantage of controlled release, where the bacteria are released gradually over time in the gut, maximizing their therapeutic potential.

Recent advances in encapsulation technology have focused on optimizing the materials used to coat probiotics, as well as refining the size and release properties of the capsules. For example, microcapsules made from alginate or chitosan can provide targeted delivery to specific regions of the GI tract, while sub-100 μm microcapsules are being developed to improve sensory properties and increase the efficiency of probiotic delivery [171]. Studies such as those by Kumherová et al. revealed that encapsulating *Bifidobacterium* strains with β-cyclodextrin combined with whey protein isolate and sodium caseinate provided excellent protection under gastric conditions [170]. Microencapsulation of *B. infantis* CCUG 52486 using sodium alginate and dairy matrices (cow or goat milk) has also shown promise [172]. This method not only improved the survival of the bacteria during GI transit but also maintained high concentrations of probiotic cells after extended refrigerated storage. Encapsulation with cow and goat milk matrices was found to be more efficient compared with other materials, like sodium alginate alone [172]. Encapsulating probiotics in conjunction with prebiotics, such as inulin, has gained attention for its ability to promote the growth and survival of the probiotics. This approach not only ensures the protection of the probiotics but also provides a substrate for microbial fermentation, enhancing their overall efficacy [173].

Although the field of bacterial encapsulation in pediatric nutrition is still in its early stages, several research projects are underway to optimize encapsulation techniques for clinical applications. These projects focus on enhancing the stability and bioavailability of probiotics, as well as exploring new materials for encapsulation that are safe and effective for use in infants. As this technology advances, encapsulated probiotics could become a standard component of pediatric nutrition, offering a reliable and efficient way to support gut health and prevent diseases in children.

## 6. Challenges and Future Directions

Scientists worldwide are actively exploring the lifelong health implications of the infant GI microbiome. Among these investigations, a crucial question being addressed is the following: what factors contribute to the variability in Bifidobacterium colonization patterns among infants globally during the transition from birth to weaning?

Despite the recognized benefits of *B. infantis*, not all infants naturally acquire adequate levels of this bacterium. Various factors, including delivery method, antibiotic exposure, and feeding practices, significantly influence the initial colonization of the infant gut. This has led to increasing interest in supplementing infant diets with *B. infantis* to ensure optimal gut health and immune development.

One major strength of this narrative review is its thorough integration of a wide range of data from both preclinical models and clinical trials, which allows for a detailed exploration of *B. infantis*’s mechanisms of action. However, this study is limited by the variability in trial methodologies and probiotic formulations, making it difficult to generalize findings. Confirmatory studies are needed to generalize findings to larger populations. Many trials had small sample sizes and inadequate randomization, affecting their ability to detect significant effects. Longer intervention periods and more definitive diagnostic tools could improve outcomes. Crossover trials need precise washout periods to avoid biases from seasonal variations. Another major limitation is that many studies are based on combinations of different probiotic strains, making it challenging to isolate and identify the specific effects of *B. infantis*. This complicates the interpretation of results, as the observed outcomes may be influenced by the synergistic or antagonistic interactions between strains, rather than the effects of *B. infantis* alone. Consequently, it is difficult to determine the true efficacy and safety of the individual strain, underscoring the need for more focused studies that examine *B. infantis* in isolation.

Advancing this field requires greater standardization in trials involving *B. infantis* strains. This requires the design of gold-standard, standardized RCTs and enhanced collaboration among research groups.

Current research focuses on understanding the best ways to supplement infants with *B. infantis* and identifying the long-term benefits of such interventions. The goal is to develop strategies that can help establish a healthy gut microbiome from early infancy, potentially reducing the risk of various health conditions later in life.

## 7. Conclusions

The studies reviewed in this manuscript suggest that *B. infantis* is more than just a resident of the infant gut; it is a vital contributor to the establishment of a healthy microbiome and the development of the immune system in infants and children. Its unique ability to efficiently utilize HMOs present in human milk provides it with a competitive advantage, allowing it to dominate the gut microbiota of BF infants and support a protective gut environment. As our understanding of the gut microbiome continues to grow, *B. infantis* stands out as a key player in promoting infant health from the very start of life. The observed decline of the *B. infantis* species in industrialized nations is a troubling trend, given its substantial impact on infant and child development. Ensuring its presence in the infant gut through breastfeeding or targeted supplementation could have profound implications for lifelong health. The demonstrated benefits of *B. infantis* in promoting gut health and preventing early childhood diseases suggest the potential for integrating specific probiotics into infant nutritional guidelines, especially for vulnerable populations such as preterm infants. Policymakers could consider endorsing *B. infantis* supplementation as part of public health initiatives aimed at improving neonatal and infant health outcomes. However, given the safety concerns raised by regulatory bodies like the FDA regarding probiotic use in hospitalized infants, further research is essential before widespread adoption.

The current evidence underscores the importance of continued research and investment into the beneficial properties of *B. infantis* strains.

## Figures and Tables

**Table 1:** Clinical studies on the GI effects of specific *B. Infantis* strains in infants and children.

Reference and Year	Study Design	N. of Subjects	Probiotic Strains	Doses/Different Concentration of Probiotic Suspensions	Effects
NEC					
Hoyos, 1999 [82]	Prospective controlled trial	2519 (2040 g average; 693M/518F probiotic group–691M/583F control group)	*B. infantis*, *L. acidophilus*	5 × 10^8^ 1 dose daily until discharge	↓ NEC-affected and fatalities caused by or associated with NEC
Bin-Nun et al., 2005 [83]	RCT	145 (VLBW; 44M/28F study group–37M/36F control group)	*B. infantis*, *B. longum*, *Streptococcus thermophilus*	1.05 × 10^9^ 1 dose daily up to the corrected age of 36 weeks	↓ NEC incidence and severity
Lin et al., 2005 [84]	RCT	367 (VLBW; 84M study group–100M control group)	*B. infantis*, *L. acidophilus*	4 × 10^9^ 2 doses daily from the 7th day until discharge	↓ NEC incidence and severity
Samanta et al., 2009 [85]	RCT	186 (VLBW)	*B. infantis*, *B. longum*, *B. bifidum*, *L. acidophilus*	2.5 × 10^9^ 2 doses daily	↓ NEC-related morbidity No placebo group included
Al-Hosni et al., 2012 [86]	RCT	101 (VLBW;22M study group–28M control group)	*B. infantis*, *L. rhamnosus*	1 × 10^9^ 1 dose daily; 28 days	↑ growth rate
Jacobs et al., 2013 [87]	Multicenter DBRCT	1099 (VLBW; 27 M study group–300M control group)	*B. infantis* BB-02, *Streptococcus thermophilus* Th2, *B. animalis* spp. *Lactis* BB12	1 × 10^9^ 2 doses daily until hospital discharge or the corrected term age	↓ incidence of NEC without a decrease in late-onset sepsis or mortality from any cause
Fernández-Carrocera et al., 2013 [88]	DBRCT	150 (VLBW)	*B. infantis*, *L. acidophilus*, *L. rhamnosus*, *L. casei, Lacticibacillus plantarum*, *Streptococcus thermophilus*	2.64 × 10^9^ 1 dose daily	↓ frequency of NEC and of the combined risk of NEC and mortality
Härtel et al., 2014 [89]	Multicenter RCT	5351 (VLBW)	*B. infantis*, *L. acidophilus*	Dose not specified 1 dose daily; 14 days	↓ risk for GI morbidity, abdominal surgery and NEC; weight gain improvement
Fortmann et al., 2020 [90]	Multicenter DBRCT	5954 (VLBW)	*B. infantis*, *L. acidophilus*	1–1.5 × 10^9^, 1–3 × 10^9^, respectively, once or twice daily, from day 1 to 3 of life until day 28	Improvement in growth in the BF group but not in the IF group; ↓ clinical sepsis in the BF group.
Robertson et al., 2020 [91]	Single-center retrospective observational study	513 (VLBW)	*B. infantis*, *L. acidophilus, B. bifidum*	1 × 10^9^ CFU of each species daily, from day 1 to 3 of life until ~34 weeks postmenstrual age	↓ incidence of NEC, ↓ late-onset sepsis, and ↓ mortality from any cause
Gastroschisis					
Powell et al., 2016 [92]	RCT	24 (>34 weeks at birth; 13M–11F)	*B. longum* ssp. *infantis* ATCC 15697	1 × 10^9^ 2 daily doses for 6 weeks or until hospital discharge	↓ *Clostridiaceae*, ↑ *Bifidobacteriaceae.* Trend towards ↓ *Streptococcaceae, Staphylococcaceae*, *Enterococcaceae*, *Enterobacteriaceae*. No effect on the duration of hospital stay.
FGID					
Dupont et al., 2010 [78]	Multicenter DBRCT	66 (3 weeks to 3 months)	IF + *B. infantis* M63, *L. rhamnosus* LCS-742 IF (controls)	10^7^ of each strain 30 days	Infants receiving M63 experienced significantly fewer feeding-related GI issues, such as vomiting, constipation, regurgitation, and flatulence.
Russo et al., 2017 [80]	Prospective RCT	55 (4–12 years; 13M and 14F for each group)	PEG + *B.infantis* M63, *B. breve* M16, *B. longum* BB536 PEG (control)	Probiotic dose not specified 8 weeks	PEG was as effective and safe with or without probiotics for treating chronic constipation in children, with no difference in effectiveness between the groups.
Giannetti et al., 2017 [79]	Multicenter RCT	73 children (8–16 years; 32M–41F)	*B. infantis* M63, *B. breve* M16, *B. longum* BB536 placebo	1 × 10^12^, 1 × 10^12^, 3 × 10^12^, respectively, 6 weeks	In children with IBS, the use of a probiotic blend was linked to improvements in abdominal pain and quality of life.
Infantile colic					
Rozé et al., 2011 [93]	Multicenter DBRCT	97 (6 months; 27F intervention group–19F control group)	IF + *B. infantis* M63, *L. rhamnosus* LCS-742, FOS IF (control)	Probiotic dose not specified 30 days	The M63 group showed ↓ crying or irritability and displayed calmer behavior (*p* < 0.02). The probiotic diet proved to be safe, easily tolerated, and effective in preventing the onset of atopic dermatitis.
Kianifar et al., 2014 [94]	RCT	45 (15–120 days; 13F/13M intervention group–12F/14M control group)	*B. infantis*, *L. casei*, *L. rhamnosus*, *Streptococcus thermophilus*, *Bifidobacterium breve, L. acidophilus*, *L. bulgaricus*, FOS	1 × 10^12^ 1 dose daily; 30 days	↓ crying time and colic
Acute diarrhea					
Vandenplas et al., 2011 [95]	RCT	11 (3–186 months; 29M/27F probiotic group–27M/27F placebo group)	*B. infantis*, *L. acidophilus*, *L. rhamnosus*, *B. animalis* spp. *lactis*, *Streptococcus thermophilus*, FOS, ascorbic acid	1.95 × 10^10^ 1 dose daily; 7 days	↓ duration of diarrhea and ↓ of prescription of further medications
Escribano et al., 2018 [62]	Multicenter DBRCT	151 term infants (< 3 months; 35M intervention group–34 M control group)	IF + *B. infantis* CECT 7210 IF (control)	1 × 10^7^ 1 dose daily; 12 weeks	In the CECT 7210 group: ↓ diarrhea episodes at week 8 along with a lower incidence of constipation. No differences were noted in other GI symptoms and growth.
Abdulah et al., 2024 [96]	RCT	101 (1.7 years; 30M/21F probiotic group–25M/25F probiotics + zinc group)	*B. infantis*, *L. paracasei*, *L. rhamnosus* *Probiotics plus zinc*	10 × 10^6^ 1 dose daily; 7 days	Probiotics plus zinc group: did not significantly impact disease severity but faster recovery times (1.34 days vs. 2.00 days, *p* < 0.001). Both groups: significant ↓ in dehydration severity and disease scores.
Ulcerative colitis					
Miele et al., 2009 [97]	DBRCT	29 (1.7–16.1 years; 13F/16M)	VSL#3 *	4.5 × 10^11^−1.8 × 10^12^ (age-dependent) 1 year	Significant efficacy for inducing and maintaining remission.

↑, increased of; ↓, reduction of; *B., Bifidobacterium*; CFU, colony forming unit; DBRCT, double-blind randomized controlled trial; FOS, fructo-oligosaccharide; GI, gastrointestinal; IF, infant formula; *L., Lacticaseibacillus*; NA, not applicable; NEC, necrotizing enterocoliits; PEG, polyethylene glycol; RCT, randomized controlled trial; VLBW, very low birth weight; * VSL#3: *B. infantis, B. breve*, *B. longum*, *Lacticaseibacillus delbrueckii* subsp. *Bulgaricus, Lacticaseibacillus acidophilus*, *Lacticibacillus plantarum, Lacticaseibacillus paracasei*, *Streptococcus salivarius* subsp. *thermophilus.*

**Table 2:** Clinical studies on the extraintestinal effects of specific *B. infantis* strains in infants and children.

Reference and Year	Study Design	N. of Subjects	Probiotic Strains	Doses/Different Concentration of Probiotic Suspensions	Effects
Autism spectrum disorder					
Sanctuary et al., 2019 [145]	Pilot cross- over RCT	11 (2–11 years; 9M–2F) ASD and GI co-morbidities	BCP *+ B. infantis* UCD272 versus BCP alone	4 × 10^9^ CFU twice daily; 5 weeks	↓ GI symptoms; ↓ occurrence of particular aberrant behaviors; well-tolerated
Wang et al., 2020 [146]	RCT	26 (4–5 years; 24M–2F)	*B. infantis* Bi-26, *L. rhamnosus* HN001, *B. lactis* BL-04, *L. paracassei* LPC-37, *FOS*	1 × 10^10^ 1 dose daily; 108 days	↑ beneficial bacteria when compared with baseline; ↓ levels of suspected pathogens; ↑ SCFA and homovanillic acid; significantly ↓ serotonin. Improved GI autism severity.
Respiratory Health and Seasonal Allergies					
Cazzola et al., 2010 [147]	Pilot RCT	135 (3–7 years), 73 placebo group (39M–34F) 62 Synbiotic group (33M–29F)	*B. infantis* R0033, *B. bifidum* R0071, *Lactobacillus helveticus* R0052, FOS	3 × 10^9^, 750 mg once daily; 3 months	↓ number of children who experienced at least one winter illness by 25%, ↓ number of school days missed
Miraglia Del Giudice et al., 2017 [148]	DBRCT	40 (9 ± 2.2 years, 18M–22F)	*B. longum* BB536, *B. infantis* M-63, *B. breve* M-16V	3 × 10^9^,1 × 10^9^, 1 × 10, respectively, once daily; 8 weeks	Significantly relieved nasal symptoms of allergic rhinitis; improved quality of life.

↓, reduction of; ↑, increased of; BCP, bovine colostrum product; *B., Bifidobacterium*; DBRCT, double-blind randomized controlled trial; FOS, fructo-oligosaccharide; GI, gastrointestinal; SCFA, short-chain fatty acids.

## Data Availability

Data sharing is not applicable to this article as no new data were created or analyzed in this study.
